# COVID-19: systematic learning across boundaries requires a conceptual agreement

**DOI:** 10.1177/14034948211027086

**Published:** 2021-06-24

**Authors:** Knut Ole Sundnes, Geir Sverre Braut

**Affiliations:** 1 The Intervention Centre, Oslo University Hospital, Norway; 2 University of Stavanger, Centre for Risk Management and Societal Safety, Norway; 3 Research Department, Stavanger University Hospital, Norway

**Keywords:** COVID-19, research, learning, Utstein-template

## Abstract

The COVID-19 epidemic has revealed a shortage of basic knowledge and understanding of pandemics, especially regarding their dynamics and how to contain them. The results are a host of governments’ decrees and instructions, one replacing the other, often within the same week. It has further, in a truly short time, resulted in an overwhelming number of publications, many of them prioritising early publication over quality. This commentary addresses the concept of structured research related to disasters and how the use of endorsed guidelines will facilitate well-designed evaluation research with improved rigour and external validity, even if applied retrospectively. The outcome should be a solidified knowledge base. Further, the important role of public health efforts is to be highlighted, as their role has proved crucial during the COVID-19 pandemic.

## Introduction

We are now facing a pandemic of historical dimensions. More than one year has passed since the first COVID-19 case was reported by China to the international community, 31 December 2019. Since then, the pandemic has taken disastrous proportions and, by end-May 2021, the reported cases sum up to almost 170 million, having claimed over 3.5 million deaths worldwide [1].

The COVID-19 pandemic has challenged the global society, more than most people and authorities had anticipated. This is regardless of nation, profession and position. Governments worldwide have been forced to instigate serious restrictions and activities to contain it, and questions concerning governance and public health have come to the forefront at an unprecedented scale. In a significant number of countries, even the somatic healthcare systems have been forced to apply triage principles; that is, rationing care for those individuals more likely to survive, curtailing medical care for other groups of patients. Nevertheless, COVID-19 has revealed that the management of this pandemic to a large extent has been trial and error, simply because the knowledge base to lean on did not exist. This has been demonstrated through the large number of decrees and instructions from the respective governments, many of them modified and adjusted on a weekly to monthly basis. The need for more structured learning processes is also emphasised in current medical publications [2].

The aim of this commentary is to point out a direction of how to solidify the research process and to establish science with acceptable internal rigour and with external validity. Never have we experienced a situation which more clearly demonstrates the role of all societal structures as this pandemic has involved all parts of society; that is, governance, including strategic and tactical initiatives, and the outcome on practically all functions of society, both private and public enterprise, and their dependencies and interdependencies. And seldom has the role of public health become more obvious.

Caused by the ultra-mobility of the global population, hardly any place can avoid this virus. Being spread globally at a higher speed than ever witnessed before, time for preparation has been limited. Further, more than any epidemic before, COVID-19 has revealed a lack of uniform understanding and management, both regarding when to define and declare a pandemic and how to handle it [3]. Decisions have been made and regulations and precepts, with significant impact on societies worldwide, have been instigated, often at short notice.

COVID-19 has shown that there are dependencies, directly and indirectly, between the disease burden imposed on the population, society’s infrastructures and the medical and social measures taken. This is acknowledged even in societies with a well-developed health system [4]. There is a need for an agreement on how such factors can be described in an unambiguous way – for example, when setting measurable targets and benchmarks for preparedness and response capacities [5]. This article will draw attention to the Utstein guidelines for disaster research as an existing framework that is possible to use in this effort [6].

## Science

Combining a shortage of substantiated knowledge on how to handle a large-scale epidemic and the principal difficulties producing science of disasters, COVID-19 has created an academic challenge seldom seen. But the situation is not unique. For a long time there has been a shortage of research and evaluation on disasters and their management in general. Not least the knowledge about the societal effects of medical emergencies seems to be poorly understood. The COVID-19 pandemic has impressively demonstrated that this is even more so for large-scale epidemics and pandemics. Even though many national health administrations around the world for two decades have been planning for possible upcoming pandemics, the plans made seem to have ignored how the broader society could be affected by such incidents.

Pandemics alongside with other disasters are suffering from insufficient science as their pure nature prohibits most forms for a structured prospective approach on research. Consequently, we are relying on empiric experience going way back in history, even before micro-organisms were discovered. Decrees issued today have a resemblance to the recommendations issued by the Prophet Mohammad in 620 AD and the royal decree of King Christian 4th of Denmark-Norway in 1625 [7, 8]. Many of these non-pharmaceutical measures seem quite sensible, but the problem is that we still do not know much about their efficacy.

The large variety of types and lengths of governmental instructions and decrees demonstrates the volatile foundation for decision making. During public debates on COVID-19 even competent persons in key positions demonstrate firm positions on many issues as if they are self-evident but, in reality, they are not necessarily substantiated. Even within the somatic hospital healthcare of today, the health authorities have stated that their overall recommendations rely on a rather unsubstantiated knowledge base [9]. For the public health aspects, this is even more so.

The wide diversity of approaches has resulted in very different immediate outcomes, not only between countries, but also within countries. In the wake of this pandemic, it is to be hoped that sociologists, public health experts, infectious disease experts and intensive care specialists will produce a series of evaluations and research to prepare us better for the next pandemic. This will require that all scientists involved take advantage of existing systems for uniform reporting, applying the same scientific tools. This way only will we be able to produce better science and draw better conclusions, regardless of from which platform they are viewing this pandemic. The Utstein guidelines for disaster research comprise such a system.

The shortage of science-based knowledge was also demonstrated by the sudden ‘tsunami’ of publications available already after 3–4 months. Taking full advantage of this special situation, both authors and journals worldwide have taken short cuts to be first with novel information. With ultra-short peer review time the necessary quality control seemed bound to fail, which also inevitably has happened. Even renowned journals have cut corners to be in the frontline. Consequently, there is reason to question both their rigour and external validity, and many papers do not meet acceptable academic standards. The result has been all shades of quality such as from blunt fraud to insufficiently substantiated new paradigms. Retraction Watch has identified 63 publications which have been retracted, the infamous ‘Surgisphere’ paper being the best known [10]. There are good reasons why the *Lancet* published an editorial under the heading ‘COVID-19: A stress-test for trust in science’ [11].

## Learning from disasters

‘Why have we not learned from what we have learned?’ This pertinent question was asked by David Nabarro from the World Health Organization (WHO) at the Phuket Conference in 2005 after the Indian Ocean tsunami? Disasters always raise questions. Not all of them can be given a substantiated answer.

Medical science, as we know it today, is very young in a historical perspective. Unfortunately, scientific rigour was developed to meet the requirements of somatic medicine, facing individuals more than dealing with populations. For disasters, this rigour was beyond reach and, consequently, unsubstantiated conclusions were many. Dr Claude de Ville de Goyet from the Pan-American Health Organization (PAHO) addressed these challenges several times – for example, in his paper ‘Stop propagating disaster myths’ [12]. Unfortunately, public health faced many of the same research challenges until the landmark book by Edward Suchman, ‘Evaluative research’, in 1967 opened a new era on evaluation [13].^1^ To scientific methodology, which for decades was synonymous with quantitative research, slowly was added methodology defined as qualitative research and, lately, also the concept of ‘mixed methods’ emerged [14]. Combined with ever stronger concepts of qualitative research, instruments to do science on and within disasters seemed doable. Unfortunately, the matrix to address this rather complex issue was still absent and, consequently, reproducible research was still a distant wish. However, since the beginning of this millennium, the Utstein guidelines for evaluation and research on disasters should have the potential to close this gap.

The COVID-19 pandemic has produced a novel, unique situation. It has not merely given a possibility, but also an obligation to address the role of governance and public health in pandemic management. If stakeholders and scientists would agree on the same matrix and templates, more solid reproducible knowledge should be extracted from this pandemic. However, the learning process from this pandemic will take time, as notes, narratives and data require proper scrutiny and analysis. Evaluation research must address both medical aspects, public health and overall governance. To foster external validity, agreed upon tools for analysis and evaluation are also crucial. One objective of this commentary is therefore to draw your attention to an already published tool to facilitate structured disaster research, which also fathoms epidemics as they are disasters in the true meaning of the word.

Core key points to address would be:

1. Public health issues and challenges
  - Identification of origin of the virus
  - Define thresholds for declaring a pandemic
  - Containment procedures
    i. Type
    ii. Duration
2. Medical treatment of mass influx of infectious disease patients reaching disastrous proportions
3. Prioritising within the healthcare system, triaging between other patient categories
4. Order of priority for vaccinations
  - Vulnerable groups, any category or
  - potential vectors; that is, mobile healthy population (to prevent spreading of disease)
    i. Healthcare workers
    ii. Teachers
    iii. Adolescents
    iv. Others
5. Identify cause–effect relationships for societal consequences outside health and care services
  - Dependencies
  - Interdependencies.

For reasons not necessarily endorsed by the authors, concomitant observation and participating observation has been viewed as both unscientific and unethical. Further, the Declaration of Helsinki demanding informed consent from patients before participating in any research programme would be an absolute ‘show-stopper’. This pandemic has, more than any incident since the Spanish flu, underlined the need for producing science concomitantly with the ongoing incident and then change the course of action if so needed [15]. This, however, requires discipline from all scientists involved and does not mean that scientific rigour and quality control take a back seat to early publication.

## The way ahead

### The Utstein concept

The ‘disaster society’ has, since mid-1990, taken action to move from the concepts of narrative reports to the production of reproducible disaster science. This required an endorsed disaster research system. Such a system must fathom an agreed terminology, an agreed generic algorithm and an agreed deconstruction of societal elements and processes, both how to prevent disasters, but also how to manage them after they materialise.

In 1994 this task was taken on by a self-established group, the Task Force on Quality Control of Disaster Management (TFQCDM). Initiated by the Nordic countries the group comprises key members from all continents, combining field experience and academic insight [6, 16]. In collaboration with the World Association for Disaster and Emergency Medicine and the Nordic Society for Disaster Medicine, and in consultations with WHO and UNOCHA, this group developed a new tool for structured disaster research: the ‘Health disaster management. Guidelines for evaluation and research in the Utstein style’. So far this has resulted in three books plus one in writing.

The absence of an agreed language forced the group first to address the challenge of concepts and language. An agreed language is crucial, not only for research, but also for cross-professional and cross-border cooperation and execution of decisions [17]. For example, it was realised that the term ‘disaster medicine’ was too narrowly associated with traumatology and somatic medicine. Therefore, the term ‘health disaster’ was introduced and strictly used. ‘Health disaster’ fathoms all mechanisms of impact on society and their resultant societal dysfunctions that also indirectly result in impaired health.

Second, the generic algorithm resulting in a disaster was defined. This Utstein algorithm describes disaster phases by their property and not by time; namely: (a) an identified hazard/threat; (b) the risk of its release; (c) actuation into an event; (d) creating structural damage (physical structures and living beings); (e) resulting in societal dysfunction; (f) prompting interventions (relief (temporary) and recovery (permanent)); (g) resulting in change; and finally (h) restoring to the pre-event societal situation. This algorithm (template) serves as the basis for the Utstein guidelines for disaster research, belonging to the ‘family’ of Utstein templates, which started with the Utstein template for cardiac research from 1991, revised in 2013 [18, 19].

Third, identifying a societal matrix comprising all basic societal functions and their interlinkage came next. Provided researchers adhere to such a system, this not only opens for reproductive research of similar events, it also opens for comparative research of generic features common to all disasters and their management. These functional and structural components are addressed in detail in the two books ‘Health disaster management. Evaluation and research in the Utstein style. Conceptual framework’, and ‘Health disaster management. Evaluation and research in the Utstein style. Structural framework. Operational framework and preparedness’ [6, 16].

Using this structure and the concise language of the Utstein guidelines, COVID-19 pandemic research should avoid confusion between scientists and produce science with acceptable rigour.

## Special features of epidemics

For most health disasters the hazard is identified or somehow anticipated. The risk of release, however, is not. When it is activated, the event itself is easy to identify and, depending on delay between identifying the event and impact on society, may give some time for preparation. Sometimes the hazard is not properly identified, but the event is immediately recognised. The recent landslide in Gjerdrum, Norway, 30 December 2020, killing 10 persons, serves as an example of the latter.

Pandemics/epidemics, however, are conceptually special. They follow the same algorithmic pattern. However, it is the damage (to living beings) and the consequent magnitude of societal dysfunction that first raise the awareness of the society. That is, the event is retrospectively identified! The origin of the hazard, however, often evades identification. If the origin is not identified or even not discussed, it will be difficult to conclude how further similar events could be prevented. However, analysing the COVID-19 event as it unfolds, the differences in societal preparedness and management should be open to solid evaluation and research, provided the different groups adhere to the same language and the same scientific concepts.

In this respect the Nordic countries have an advantage. As societies they are quite similar. Nevertheless, they are different cohorts with their specific features. Further, as there are differences in how they have addressed this pandemic, there should be a lot of lessons to learn, provided the scientific methodology is the same. What will be the immediate short-term outcome of the different actions taken? But what may be more important, what will be the long-term outcome of this pandemic and the respective actions taken? The concepts as outlined with the ‘logical framework approach’ are important as the focus should be on both short and long-term evaluation of COVID-19 [6].

The Utstein concept of data gathering and subsequent evaluation is well suited for fine-tuning with novel models for learning from practical handling of emergencies [17]. In particular, it should be acknowledged that the Utstein template is designed to demonstrate the interconnections between medical knowledge, provision of health services and societal phenomena and effects.

## Conclusion

More than anything, this COVID-19 pandemic has demonstrated the importance of endorsed guidelines also for disaster research and the importance of avoiding short cuts on quality control (the peer reviewing process).

The COVID-19 pandemic has revealed a shortage of knowledge necessary to guide health authorities on how to manage pandemics resulting in trial and error how to contain it. In an attempt to close this gap, the pandemic has resulted in numerous scientific publications. Unfortunately, both authors and journals seem to have cut corners. The result is a lack of scientific rigour reducing the possibility to make valid comparisons. Consequently, they do not bring reliable answers to the questions asked. As a result, governance of the pandemic, in real time, becomes difficult.

This pandemic should have the potential to foster scientific rigour into the ‘science of medical uncertainty’. Abiding by the ‘laws’ of science, and adhering to research tools developed for the sole purpose of strengthening the scientific rigour in disaster evaluation research, could change this for the better and make us better prepared for the future. However, science takes time. Short cuts for the sake of early publication should have no room. Standard procedures and concepts for scientific rigour, combined with applying a standardised matrix and language, will improve validity, both internal and external, for the benefit of the future. ‘Cutting corners’ regarding quality control and normal procedures will not bring knowledge to the table.

Pathogens have always been there. They have had a profound influence on history, defined the course of politics and even eradicated entire populations. As stated by William H. McNeill in his landmark book ‘Plagues and peoples’ in 1976:

In an effort to understand what lies ahead, as much as what lies behind, the role of infectious disease cannot properly be left out of consideration. Ingenuity, knowledge, and organization alter, but cannot cancel humanity’s vulnerability to invasion by parasitic forms of life. Infectious disease which antedated the emergence of humankind will last as long as humanity itself, and will surely remain, as it has been hitherto, one of the fundamental parameters and determinants of human history [7].

We need to learn! The lists of questions are many. Fortunately, today we are in a better position to find their answers. There are, however, no short cuts to wisdom. Can the Nordic countries, being so similar but nevertheless so different, contribute to this endeavour, especially if they unite behind the structured guidelines? These countries could be an epidemiological laboratory for developing models for better understanding the intersection between societal actions based on medical knowledge and their subsequent effects on society at large.

## Notes

1 This book by Edward Suchman was reprinted nine times as from 1968 to 1976 without being edited.
